# Generation of a *C. elegans tdp-1* null allele and humanized *TARDBP* containing human disease-variants.

**DOI:** 10.17912/micropub.biology.000693

**Published:** 2023-06-06

**Authors:** Jeremy Lins, Trisha J Brock, Chris E Hopkins, Anne C Hart

**Affiliations:** 1 Department of Neuroscience, Brown University, Providence, RI 02912; 2 InVivo Biosystems, Eugene, OR, 79402

## Abstract

Clinical variants of *TARDBP* are associated with frontotemporal dementia (FTD), amyotrophic lateral sclerosis (ALS) and other degenerative diseases. The predicted *C. elegans* ortholog of *TARDBP* is encoded by *tdp-1* , but functional orthology has not been demonstrated *in vivo.* We undertook CRISPR/Cas9-based genome editing of the *tdp-1* locus to create a complete loss of function allele; all *tdp-1* exons and introns were deleted, creating *tdp-1(tgx58)* , which resulted in neurodegeneration after oxidative stress. Next, we undertook CRISPR-based genome editing to replace *tdp-1* exons with human TARDBP coding sequences, creating humanized ( *hTARDBP* ) *C. elegans* expressing TDP-43 *.* Based on the efficiency of this genome editing, we suggest that iterative genome editing of the *tdp-1* target locus using linked coCRISPR markers, like *dpy-10* , would be a more efficient strategy for sequential assembly of the large engineered transgenes. *hTARDBP* decreased the neurodegeneration defect of *tdp-1(tgx58)* , demonstrating functional cross-species orthology. To develop *C. elegans* models of FTD and ALS, we inserted five different patient *TARDBP* variants in the *C. elegans* *hTARDBP* locus. Only one clinical variant increased stress-induced neurodegeneration; other variants caused inconsistent or negligible defects under these conditions. Combined, this work yielded an unambiguous null allele for *tdp-1* , a validated, humanized *hTARDBP,* and multiple ALS/FTD patient-associated variant models that can be used for future studies.

**Figure 1. Humanizing tdp-1 to determine if patient variants cause stress-induced neurodegeneration:**
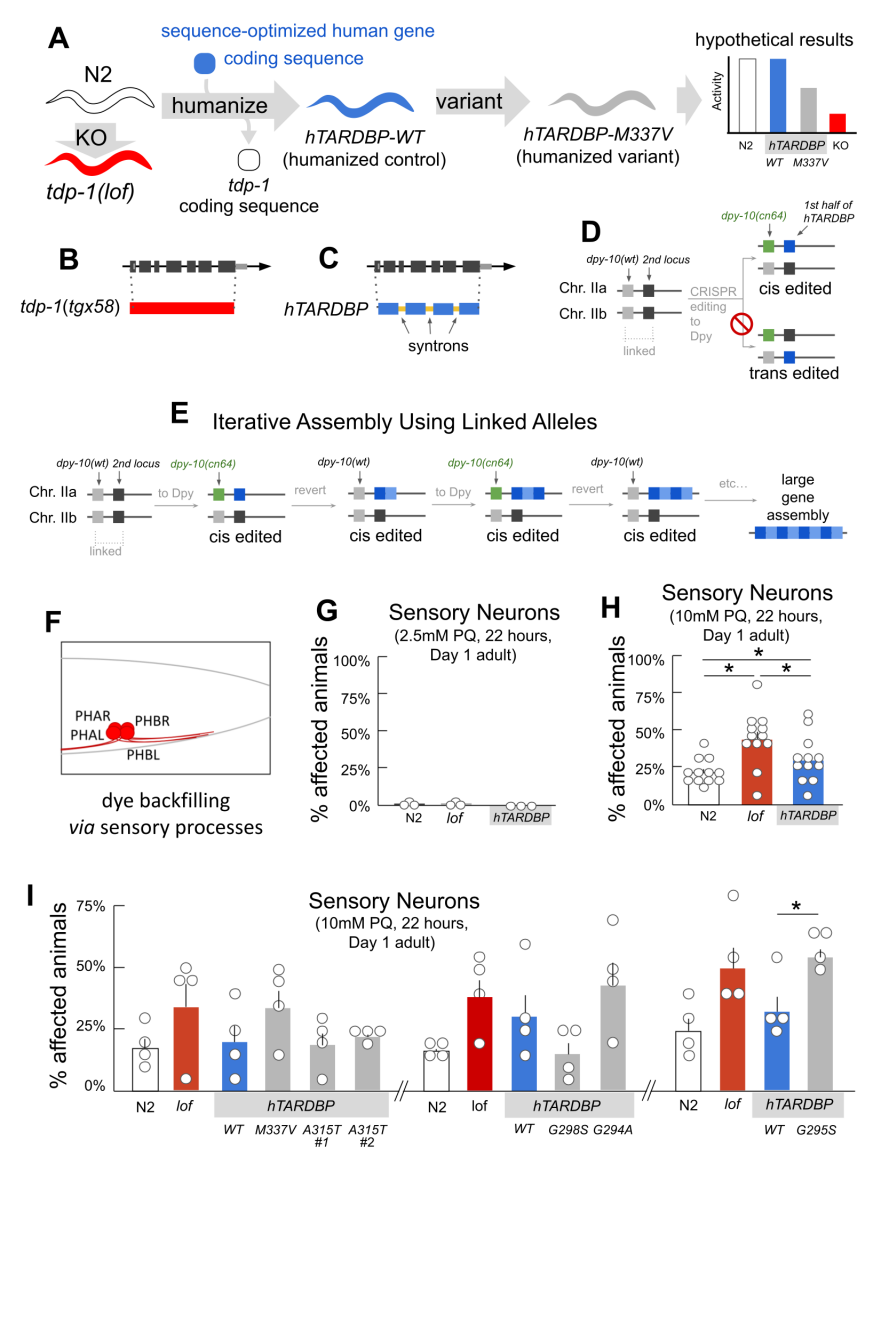
**A. Strategy for genome editing.** A wild type (N2, white) animal is CRISPR-edited to make a knock-out (KO, red) animal. When loss-of-function defects are detected in the KO, the gene is humanized (blue); wild type *C. elegans* exons and introns are excised and replaced with human coding sequences. Finally, another round of CRISPR-editing is undertaken to insert clinical variants (grey) into the humanized locus. Comparison of the N2 and KO permits detection of loss of function defects. Comparison of these with the humanized-control reveals functional orthology by rescue, and comparison to patient-associated allele lines allows detection of variant defects. **
B. Knock out of *tdp-1* locus *.*** Creation of a *tdp-1* KO null allele by CRISPR-editing required deleting sequences (red) containing exons and introns (grey boxes and intervening lines). **
C. Human gene replacement at *tdp-1* locus.** CRISPR-editing was used to remove endogenous coding sequences and insert human gene coding sequences to create *hTADRBP* , containing artificial introns (blue exon boxes linked by orange artificial intron line). **D. Cis editing predominates.** A CRISPR-editing approach was used to insert hTARDBP sequences into the *tdp-1* locus (black), which is closely linked to the *dpy-10* locus (grey). These loci are ~2 centiMorgans apart. At the co-CRISPR marker *dpy-10* locus, one allele was converted to *dpy-10* ( *cn64* ) and the other allele was unedited (hemi-converted); the resulting Roller heterozygotes were selected (green). Co-conversion of the *tdp-1* target locus with hTARDBP fragment insertion occurred only on the cis strand (blue). **E. Iterative assembly using linked alleles.** Target genes that are tightly linked to a coCRISPR marker can be iteratively co-CRISPR-edited to build large transgenes by flip-flop selection procedure. In this example, the *dpy-10* locus is co-CRISPR converted to yield *dpy-10* ( *cn64* ) (green) and conversion of the linked locus to contain a gene fragment content (dark blue) in the first step. In the second step, using the Dumpy animals from the first step, a repeat round of co-CRISPR restores *dpy-10* ( *wt* ) (grey) simultaneously with insertion of another gene fragment (light blue). Repeat rounds of co-CRISPR using the Dumpy and nonDumpy phenotypes allows iterative editing of the target locus and efficient sequential build up of a large gene assembly (additional blue boxes). **F. Assessing neurodegeneration** *C. elegans* phasmid neurons take up fluorescent lipophilic dyes via exposed sensory endings. Sensory ending defects, process retraction or neuron loss can cause dye-filling defects. **G. Mild stress** with 2.5mM paraquat for 22 hours does not cause dye filling defects in N2 (white), WT *hTARDBP* (HA3971, blue), or *tdp-1(-)* animals ( *lof,* HA3703). Three independent trials; 20 animals per genotype per trial. **H. Moderate stress** with 10mM paraquat for 22 hours causes dye filling defects in *tdp-1(tgx58)* loss of function animals *(lof* , HA3703, red) that exceed defects observed in N2 animals (white) or WT *hTARDBP* animals (HA3971, blue). Twelve independent trials; 20 animals per genotype per trial. By student’s T-test, p value for *tdp-1(lf) vs* N2 is 0.001 and *vs hTARDBP* is 0.056, while *hTARDBP vs* N2 is likely not significant at 0.11. **
I. Patient variants inserted into *hTARDBP*** Generation of patient variants M337V, A315T, G298S, and G294A did not result in moderate stress-induced dye filling defects, only G295S differed from *hTARDBP* . Three independent trials with 20 animals per genotype per trial; hash marks on X-axis group separate genotypes assayed simultaneously. Note that the experimental results shown in Panel G for N2, *hTARDBP* and *tdp-1(-)* are the same experimental results as shown in Panel F; results are consolidated in Panel F for ease of presentation. Strains used were HA4008 for M337V, HA4006 for A315T#1, HA4007 for A315T#2, HA4003 for G298S, HA4005 for G294A, HA3983 for G295S. By student’s T-test, p value for *WT* versus G298S is <0.05.

## Description

Patient variants in *TARDBP* , which encodes the RNA-binding protein TDP-43, are associated with a wide variety of degenerative disorders. These include frontotemporal dementia (FTD) which leads to degeneration and death of cortical neurons, as well as amyotrophic lateral sclerosis (ALS), which leads to degeneration and death of spinal and cortical motor neurons. The *C. elegans* ortholog of *TARDBP* is *TAR DNA-binding Protein homolog-1* , known as *tdp-1* . To enable studies of TDP-1 function, and to demonstrate functional homology between TDP-1 and TARDBP, as well as to examine how patient-associated missense variants alter protein function in live animals, we undertook several rounds of CRISPR/Cas9-based genome deletion, humanization, and variant generation (Panels A, B and C), with a focus on generating reagents and assessing neurodegeneration.

**Knock out.** First, we created a *tdp-1* loss of function allele by precise excision using CRISPR-editing. Using CRISPR/Cas9-mediated homologous recombination and *dpy-10* co-conversion (Arribere et al. 2014) , we designed a knockout (KO) that would remove all *tdp-1* coding exons, as well as introns (Panel B) resulting in an unequivocal complete loss of function allele. When generating *tdp-1* KOs *,* we observed robust co-conversion of the *dpy-10* and *tdp-1* loci on the same maternal chromosome (Panel D), as expected from previous work (Medley et al. 2022) . However we also observed biallelic conversion of the sister chromosome, as seen previously (Kim et al. 2014) , which allowed us to isolate the *tdp-1(tgx58)* deletion as homozygote, free of the Dumpy phenotype marker.

**Assess neurodegeneration for *tdp-1(lof).*** To determine if loss of *tdp-1* function in *C. elegans* leads to degeneration and/or loss of neurons, we examined stress-induced degeneration of glutamatergic sensory neurons. PHA and PHB neurons are bilaterally-symmetric phasmid neurons in the *C. elegans* tail that have exposed sensory endings (Panel F). Degenerative retraction of phasmid neuron sensory processes or phasmid neuron death can prevent fluorescent dye back-filling of the entire neuron. This assay has been used previously in *C. elegans* models of neurodegenerative disease (Faber et al. 1999; Baskoylu et al. 2018; Ryan et al. 2020) . Hypersensitivity to stress can reveal latent defects in *C. elegans* models of neurodegenerative disease (Baskoylu et al. 2018; Nass et al. 2002) . Noting reports that perturbation of *tdp-1* function causes hypersensitivity to oxidative stress (Vaccaro, Tauffenberger, Ash, et al. 2012) , we compared wild type animals to *tdp-1(tgx58)* animals at two concentrations of paraquat. Mild paraquat stress overnight had no impact on neurodegeneration in the dye filling assay in either wild type or *tdp-1(tgx58)* young adult animals (Panel G, 22 hours, 2.5mM paraquat). However, 10mM paraquat-treatment overnight led to modest dye filling defects in wild type with more dramatic defects in *tdp-1(tgx58)* animals (Panel H, 22 hours, 10mM paraquat). These results confirm that *tdp-1* loss of function increases neurodegeneration under moderate oxidative stress.

**Humanize and insert clinical variants.** With a KO phenotype established, we used two serial rounds of co-CRISPR editing (Arribere et al. 2014) to insert sequences encoding human *TARDBP* as a replacement of the *tdp-1* coding sequence (Panel C). For this humanized construct, we focused on expressing the most abundant isoform of TARDBP (UNIPROT Q13148-1) re-coded for *C. elegans* codon bias and with the insertion of three synthetic introns. A full length plasmid containing a synthetic TARDBP sequence could not be generated by our suppliers. As an alternative, we elected to consecutively insert two fragments of TARDBP using co-CRISPR-editing. Insertion of the first fragment of recoded TARDBP resulted only in cis co-editing events (target edit occurring on same strand as selection marker, Panel D); all animals homozygous for the intended *tdp-1* edit were also Dumpy (homozygous for *dpy-10(cn64)* ). To move *hTARDBP* to a wild type background, we undertook an additional round of CRISPR-editing to restore the original N2 wild type *dpy-10* sequence ( *dpy-10(wt)* ). Another round of co-CRISPR to insert the second fragment of recoded TARDBP also resulted in only cis co-editing and all animals homozygous for full length TARDBP were also Dumpy. Again, a round of repair CRISPR was used to restore the *dpy-10* locus back to nonDumpy (original N2 sequence, wild type phenotype).

The observation of cis-only editing with the co-generation of *dpy-10* alleles, followed by a need to repair to get back to wildtype phenotype, leads us to suggest that future studies should use a serial-editing strategy to build up a large gene with a “flip-flop approach” that alternates between Dumpy and nonDumpy animals (Panel E).

Expression of full length *hTARDBP* was confirmed by rtPCR and the LOF defect of *tdp-1(-)* was partially rescued in the *hTARDBP* animals. (Panel H). We used co-CRISPR editing to insert each of the five ALS/FTD clinical variants into the humanized locus (G294A, G295S, G298S, A315T, and M337V). Over-expression of TARDBP A315T is toxic in two different *C. elegans* models and TARDBP M337V is toxic in two different *C. elegans* models (Vaccaro, Tauffenberger, Aggad, et al. 2012; Liachko, Guthrie, and Kraemer 2010; Zhang et al. 2012) . *C. elegans* models for the other alleles have not been reported previously.

**Assess neurodegeneration for clinical variants.** Only insertion of *hTARDBP-G295S* yielded a significant defect versus *hTARDBP-WT* (Panel I). Future work will likely require confirmation of the G295S defect with the generation and testing of another independently-derived allele. For the remaining alleles (G294A, G298S, A315T, and M337V), no significant increases in stress-induced neurodegeneration were observed, compared to *hTARDBP-WT* , using this assay.

## Methods

Neurodegeneration: L4 stage animals were moved to NGM/OP50 plates containing 10mM (or 2.5mM) paraquat for 22 hours and neurodegeneration was examined the next day in adults based on backfilling of phasmids neurons with DiI (Molecular Probes). Animals were scored as affected if any of the four phasmid neurons failed to backfill with fluorescent dye, detected at a magnification level of 12.5x with moving animals on culture dishes; note that loss of a single neuron will be missed in some animals and we may underestimate neurodegeneration. Animals were scored blinded as to genotype.

Genome editing: The *tdp-1(tgx58)* KO allele was generated by deletion of the coding sequence using CRISPR/Cas9-mediated homologous recombination. The *dpy-10* co-conversion strategy was used to identify candidate lines after injection (Arribere et al. 2014) . Two guide RNAs (sgRNAs) were selected, one in the first *tdp-1* coding exon and the other after the stop codon creating a 1787bp deletion. All sgRNAs were synthesized by Synthego Corporation (Redwood City, CA). The donor homology sequence was a single stranded oligonucleotide (ssODN) containing 35bp homology arms, a 3-frame stop sequence, and an XhoI restriction site (reagent table). Injections of the Cas9 protein, sgRNAs, and donor homology template mix were performed with N2 young adults. Genome edit candidates were selected from the F1 population based on a visual screen for the co-conversion Rol phenotype and screened by PCR for the deletion. Homozygous deletion lines were confirmed by sequencing.

The *hTARDBP* - *WT* insertion was generated using a gene swap method (McCormick et al. 2021) with modifications to use PCR products as the donor homology template through sequential rounds of editing. The first insertion used the *dpy-10* co-conversion CRISPR/Cas9 strategy to insert 725bp of the *hTARDBP* sequence. The injection mix included Cas9 protein, the 795bp dsDNA donor homology template, sgRNAs, and co-CRISPR reagents. This was injected into the gonads of adult hermaphrodite worms and the F1 animals displaying the co-CRISPR phenotype were isolated. Homozygous animals containing the insertion were identified; however, they also contained the *dpy-10(cn64)* mutation. An edit to correct this mutation was performed and animals with wild type sequence at the *dpy-10* locus, but containing the partial *hTARDBP-WT* were identified. This strain was injected with the 756bp PCR product donor homology template containing the second half of the *hTARDBP* sequence and homology arms, along with the Cas9 protein, sgRNAs, and the *dpy-10* co-CRISPR reagents. Animals homozygous for the insertion as well as the *dpy-10(cn64)* mutation were isolated and the corrective edit for *dpy-10* was performed. Integration of the complete sequence of *hTARDBP* was confirmed by Sanger sequence analysis. When complete, the *hTARDBP-WT* strain contained a codon optimized version (Redemann et al. 2011) of 414 amino acid *hTARDBP* with 3 synthetic introns. Note that *hTARDBP* was introduced into the native *tdp-1* exon 1 such that the first 5 native *C. elegans* amino acids were preserved ( *i.e.* MADET). Verification *hTARDBP* mRNA expression was undertaken. RNA was extracted from the *hTARDBP-WT* strain and N2 using TRI Reagent and the Direct-zol RNA kit (Zymo Research) and cDNA was synthesized using iScript Reverse Transcription Supermix for RT-qPCR (Bio-Rad Laboratories). To amplify the whole coding sequence, PCR was performed, see reagent table for primers, on the cDNA and visualized on an agarose gel. The full length *hTARDBP* sequence amplified from the humanized strain at a comparable level to *tdp-1* in N2. The amplicons were also used as a template for DNA sequencing by Sequetech Corporation. Sequences were aligned and analyzed using Benchling and full-length *hTARDBP* was observed.

The *hTARDBP* variants were inserted using CRISPR/Cas9-mediated homologous recombination. For each variant, two sgRNAs flanking the desired edit were selected. The donor homology was designed with 35bp homology arms and re-coding to eliminate recutting of the repaired template and to make identification of the edits easier. The injection mixes included Cas9 protein, each donor homology single-stranded oligonucleotide (ssODN), the sgRNAs, and the *dpy-10* co-CRISPR reagents. Animals displaying the co-CRISPR phenotype were selected and High Resolution Melt Analysis was performed to identify animals containing the edit of interest. Homozygous animals were isolated and confirmed by sequencing of both the target locus and the *dpy-10* locus. Original, edited strains were backcrossed to N2 four times to create outcrossed strains, which were used to generate results presented herein.

**Statistical analysis:** Student’s t-test used for comparison of neurodegeneration (Microsoft Excel).

## Reagents

**Table:** 

**gene**	**outcrossed strain**	**origin strain**	**genotype**	**source**
*tdp-1(tgx58)*	HA3703	NMX67	*tdp-1(tgx58 [1787bp deletion])*	This study
*hTARDBP(wt)*	HA3971	NMX298	*tdp-1(tgx286 [humanTARDBP WT]*	This study
*hTARDBP(G295S)*	HA3983	NMX379	*tdp-1(tgx286tgx359 [humanTARDBP G295S]) II*	This study
*hTARDBP(G298S)*	HA4003	NMX392	*tdp-1(tgx286tgx371 [humanTARDBP G298S]) II*	This study
*hTARDBP(G294A)*	HA4005	NMX406	*tdp-1(tgx286tgx382 [humanTARDBP G294A]) II*	This study
*hTARDBP(A315T)*	HA4006	NMX440	*tdp-1(tgx286tgx416 [humanTARDBP A315T]) II*	This study
*hTARDBP(A315T)*	HA4007	NMX441	*tdp-1(tgx286tgx417 [humanTARDBP A315T]) II*	This study
*hTARDBP(M337V)*	HA4008	NMX424	*tdp-1(tgx286tgx400 [humanTARDBP M337V]) II*	This study

**Table:** 

**reference**	**sgRNA 1**	**sgRNA 2**	**ssODN sequence or plasmid name**
			
*tdp-1(-)*	AATGGCCGACGAAACGCCGA	CGTCGACATGAAAATTGTAG	ATCTGTTTCCAGTCACTAAATGGCCGACGAAACGCTAAATAAATAAACTCGAGCAATTTTCATGTCGACGCACTTTGCGATTTTATGC
*hTARDBP(wt) part 1*	​​AATGGCCGACGAAACGCCGA	CCGGTCGATCACGTTCTCTG	tatctgtttccagTCACTAAATGGCCGACGAAACGATGTCCGAGTACATCCGTGTCACGGAGGATGAAAACGACGAGCCAATCGAGATCCCATCCGAGGACGACGGAACCGTCCTCCTCTCCACCGTCACCGCCCAATTCCCAGGAGCCTGCGGACTCCGTTACCGTAACCCAGTCTCCCAATGCATGCGTGGAGTCCGTCTCGTCGAGGGAATCCTCCACGCCCCAGACGCCGGATGGGGAAACCTCGTCTACGTCGTCAACTACCCAAAGGACAACAAGCGTAAGATGGACGAGACCGACGCCTCCTCCGCCGTCAAAGTCAAGCGTGCCGTCCAAAAGgtgagttattataatttttttgatcacaacgattattttaattttcagACCTCGGACCTCATAGTGCTCGGACTCCCATGGAAGACCACCGAGCAAGACCTCAAGGAGTACTTCTCCACCTTCGGAGAGGTCCTCATGGTCCAAGTCAAGAAGGACCTCAAGACCGGACACTCCAAGGGATTCGGATTCGTCCGTTTCACCGAGTACGAGACCCAAGTCAAGGTCATGTCCCAACGTCACATGATCGACGGACGTTGGTGCGACTGCAAGCTCCCAAACTCCAAGCAATCCCAAGACGAGCCACTCCGTTCCAGGAAGgttaaatgtacaaacaactatttgaaagattttctcacccgattttttcagGTCTTCGTCGGACGTTGCACCGAGGACATGACCGAGGACGAGCTCCGTGAAGAACGTGATCGACCGGATAGACGACCGATTCAAA
*hTARDBP(wt) part 2*	TTGCACCGAGGACATGACCG	GAGACTCGAGAGGACCAGGA	tcagGTCTTCGTCGGACGTTGCACCGAGGACATGACTGAAGATGAACTTAGAGAGTTCTTCTCCCAATACGGAGACGTCATGGACGTCTTCATCCCAAAGCCATTCCGTGCCTTCGCCTTCGTCACCTTCGCCGACGACCAAATCGCCCAATCCCTCTGCGGAGAGGACCTCATCATCAAGgtaaataattatacattcgatgataaatttatgcgtactatttttcagGGAATCTCCGTCCACATCTCCAACGCCGAGCCAAAGCACAACTCCAACCGTCAACTCGAGCGTTCCGGACGTTTCGGAGGAAACCCGGGGGGATTCGGAAACCAAGGAGGGTTCGGGAACTCCCGTGGAGGAGGAGCCGGACTCGGGAACAACCAGGGATCCAACATGGGAGGAGGAATGAACTTCGGAGCCTTCTCCATCAACCCAGCCATGATGGCCGCCGCCCAAGCCGCCCTCCAATCCTCCTGGGGAATGATGGGAATGCTCGCCTCCCAACAAAACCAATCCGGACCATCCGGAAACAACCAAAATCAGGGGAACATGCAACGTGAGCCAAACCAAGCCTTCGGATCGGGGAACAACTCCTACTCCGGATCCAACTCCGGAGCCGCCATCGGATGGGGATCCGCCTCCAACGCGGGGTCCGGATCCGGGTTCAACGGAGGATTCGGATCCTCCATGGACTCCAAGTCCTCCGGATGGGGAATGTAAGGATGGTGATTCTCTCGAAAAATCGTTATTATTCA
*hTARDBP(G295S)*	GACGTTTCGGAGGAAACCCG	GCCGGACTCGGGAACAACCA	GTCAACTCGAGCGTTCCGGACGTTTCGGAGGAAACCCAGGTGGTTTCGGTAACCAGGGTGGTTTTGGCAATTCAAGGGGATCTGGAGCAGGTCTTGGTAATAACCAGGGATCCAACATGGGAGGAGGAATGAACTTCG
*hTARDBP(G298S)*	GGAACGCTCGAGTTGACGGT	GCCGGACTCGGGAACAACCA	CATCTCCAACGCCGAGCCAAAGCACAACTCCAACAGACAGCTTGAACGCTCTGGAAGATTCGGTGGTAATCCGGGAGGTTTCGGTAATCAGGGTGGTTTCGGTAACTCTAGAGGCGGTGGAGCTTCTCTTGGAAATAACCAGGGATCCAACATGGGAGGAGGAATGAACTTCG
*hTARDBP(G294A)*	GACGTTTCGGAGGAAACCCG	GCCGGACTCGGGAACAACCA	GTCAACTCGAGCGTTCCGGACGTTTCGGAGGAAACCCAGGAGGTTTTGGTAATCAAGGTGGATTTGGAAATTCTAGAGCTGGTGGTGCTGGTTTAGGTAATAACCAGGGATCCAACATGGGAGGAGGAATGAACTTCG
*hTARDBP(A315T)*	GCCGGACTCGGGAACAACCA	TTGGAGGGCGGCTTGGGCGG	AACTCCCGTGGAGGAGGAGCCGGACTCGGGAACAACCAAGGCTCTAATATGGGCGGCGGCATGAATTTTGGAACCTTTTCTATTAATCCCGCTATGATGGCTGCTGCCCAAGCCGCCCTCCAATCCTCCTGGGGAATGATG
*hTARDBP(M337V)*	TTGGAGGGCGGCTTGGGCGG	GCCAAACCAAGCCTTCGGAT	AGCCTTCTCCATCAACCCAGCCATGATGGCCGCCGCTCAGGCTGCTCTTCAATCTTCTTGGGGTATGGTTGGTATGCTTGCTTCTCAACAGAACCAGTCTGGTCCGTCTGGTAATAACCAGAACCAAGGGAATATGCAGCGCGAACCAAATCAGGCTTTTGGATCGGGGAACAACTCCTACTCCGGATCCAACTCC
*dpy-10(wt)*	CTCGTGGTGCCTATGGTAGC		CACTTGAACTTCAATACGGCAAGATGAGAATGACTGGAAACCGTACCGCTCGTGGTGCCTATGGTAGCGGAGCTTCACATGGCTTCAGACCAACAGCCTAT

**Table:** 

Primer name	Primer Sequence
hTARDBP FWD (86)	GGAAACCTCGTCTACGTCGTCA
hTARDBP REV (88)	ATCCGAATCCTCCGTTGAACCC
tdp-1 FWD (87)	CCGACGAAACGCCGAAGG
tdp-1 REV (89)	GTCTCCAGGTGCCCAGTATCTC

**Table:** 

**reagent**	**source**
1,1'-Dioctadecyl-3,3,3',3'-Tetramethylindocarbocyanine Perchlorate (DiI)	Molecular Probes
XhoI (cat#R0146L)	New England Biochemicals
Cas9 protein with NLS, high concentration (cat#CP02)	PNA Bio
TRI Reagent (cat#R2050-1-200)	Zymo Research
Direct-zol RNA Miniprep (cat#R2052)	Zymo Research
iScript™ Reverse Transcription Supermix for RT-qPCR (cat#1708840)	Bio-Rad Laboratories
