# Corrigendum: Generation of a *C. elegans tdp-1* null allele and humanized *TARDBP* containing human disease-variants.

**DOI:** 10.17912/micropub.biology.002225

**Published:** 2026-06-03

## Description

Corrigendum for microPublication Lins et al., 2023 https://www.micropublication.org/journals/biology/micropub-biology-000693

The authors of the above-noted microPublication submit the following corrections.

After publication, it was determined that the humanized-control *hTARDBP* transgene and other mutant versions of *hTARDBP* contain a duplication, which arose during the second genome-editing step. These duplications were detected using PCR primers and were confirmed by sequencing. These duplications prevent stable expression of the encoded protein.

The only *hTARDBP* transgene that was assembled correctly is in strain HA4006 called *tdp-1(tgx286tgx416)* , which contains the A315T mutation, and is accurately described in the original microPublication.

The precise deletion loss-of-function allele, *tdp-1(tgx58)* in strain HA3703, has been confirmed and is also accurately described in the original microPublication.

Conclusions have not changed regarding results in Panels G, H and I comparing the deletion allele *tdp-1(tgx58)* to wild type animals (N2). And, conclusions have not changed regarding results in Panel I, which compare *tdp-1(tgx286tgx416)* A315T to the deletion loss-of-function allele *tdp-1(tgx58)* and to wild type animals (N2 strain).

No conclusions should be drawn from results in Panels H & I regarding the mis-assembled humanized-control *hTARDBP* transgene or the other mutant versions of *hTARDBP.*

These changes have not been applied to the published article. 

